# Ambiguity Resolution for Passive 2-D Source Localization with a Uniform Circular Array

**DOI:** 10.3390/s18082650

**Published:** 2018-08-13

**Authors:** Jinlong Xin, Guisheng Liao, Zhiwei Yang, Haoming Shen

**Affiliations:** 1 National Laboratory of Radar Signal Processing, Xidian University, Xi’an 710071, China; yangzw@xidian.edu.cn (Z.Y.); shemerming@163.com (H.S.); 2 Collaborative Innovation Center of Information Sensing and Understanding, Xidian University, Xi’an 710071, China

**Keywords:** array signal processing, 2-D source localization, phase ambiguity resolution, uniform circular array

## Abstract

This paper proposes two novel phase-based algorithms for the passive localization of a single source with a uniform circular array (UCA) under the case of measuring phase ambiguity based on two phase difference observation models, which are defined as the unambiguous-relative phase observation model (UARPOM) and the ambiguous-relative phase observation model (ARPOM). First, by analyzing the varying regularity of the phase differences between the adjacent array elements of a UCA, the corresponding relationship between the phase differences and the azimuth and elevation angle of the signal is derived. Based on the two phase observation models, two corresponding novel algorithms, namely, the phase integral accumulation and the randomized Hough transform (RHT), are addressed to resolve the phase ambiguity. Then, by using the unambiguous phase differences, the closed-form estimates of the azimuth and elevation angles are determined via a least squares (LS) algorithm. Compared with the existing phase-based methods, the proposed algorithms improve the estimation accuracy. Furthermore, our proposed algorithms are more flexible for the selection of an array radius. Such an advantage could be applied more broadly in practice than the previous methods of ambiguity resolution. Simulation results are presented to verify the effectiveness of the proposed algorithm.

## 1. Introduction

Passive source localization using an array of sensors has received considerable attention for use in wireless communication, electronic reconnaissance, sonar, and other applications in recent decades [1,2,3]. Uniform circular arrays (UCAs) are extensively utilized in the context of two-dimensional (2-D) angle estimation due to their attractive advantages, including 360° azimuthal coverage, almost unchanged directional patterns, and additional elevation angle information. Many advanced algorithms have been developed for 2-D direction-of-arrival (DOA) estimation with UCAs [4,5,6,7,8].

There are two main types of solution in the context of 2-D parameter estimation, which are spectrum-based and phase-based algorithms. The spectrum-based algorithms [3,4,5,6] can obtain source localization with high precision, but they suffer from a high computational cost from the eigenvalue decomposition and spectrum search operations. To address this problem, phase-based algorithms are proposed in [7,8], which are efficient in that they are free of eigenvalue decomposition and spectrum search. However, if the inter-element spacing exceeds a half-wavelength of the source, periodical ambiguities will be introduced into the phase measurements with an ambiguity period of 2π, which will lead to inaccuracies in parameter estimation.

The major problem for source localization is to solve the directional ambiguity when using the phase-based algorithm in DOA estimation under the case of measuring phase ambiguity. The DOA estimation with an interferometer is a typical phase-based algorithm. By varying the interferometer structure instantaneously, time-varying array systems with multiple channels are developed to remove directional ambiguities [9,10,11]. In [12,13,14,15], a rotary method with rotating long-baseline interferometers is proposed to resolve ambiguity. Referring to the idea of ambiguity resolution via a rotating interferometer, the authors in [16] propose a subarray grouping and ambiguity searching method to estimate the parameters of a near-field source, which utilizes the different ambiguity properties of each subarray divided by a UCA. This method can achieve unambiguous DOA estimation. However, this method needs to search the unambiguity phase difference and is limited to the case where there is an even number of sensors and there is eight or more sensors total. Likewise, the localization algorithms of a single three-dimensional source using the UCA are developed in [17,18]. However, it is worth noting that the radii r≤λ/4 and r≤λ/(4sin(π/M)) are strictly required to yield an unambiguous source location in methods [17,18], respectively, which may be infeasible or impractical in many practical applications.

Aiming to address the problems in the existing phase-based algorithms for ambiguity resolution, we propose two novel algorithms for the localization of a single source with a UCA under the case of measuring phase ambiguity. First, the corresponding relationship between the phase differences and the azimuth and elevation angles of a signal is derived by analyzing the varying regularity of the phase differences between the adjacent array elements of a UCA. Then, two types of phase-difference observation models, namely, the unambiguous-relative phase observation model and the ambiguous-relative phase observation model, are defined according to the increment of the adjacent measured phase differences. Based on the unambiguous-relative phase observation model, a method of phase integral accumulation is applied to recover the unambiguous-relative phase differences, and the closed-form estimates of the azimuth and elevation angles are determined via a least squares (LS) algorithm. Furthermore, based on the ambiguous-relative phase observation model, the randomized Hough transform (RHT) algorithm is first utilized to detect the parameters of the zero-baseline phase-difference curve, which we call the coarse estimates of the azimuth and elevation angles of the signal. Then, with the coarse 2-D DOA estimates, the unambiguous phase differences can be obtained, and the fine angle estimates are determined by the LS algorithm. Compared with the existing phase-based methods, the proposed algorithms can improve the estimation accuracy and are more flexible for the selection of an array radius. Such an advantage could be applied more broadly in ambiguity resolution, and has further applications for unambiguous direction finding in a very wide frequency band.

The rest of the paper is organized as follows. Section 2 introduces the 2-D DOA estimation model of a narrowband source. Section 3 provides two novel unambiguous DOA estimation algorithms under the case of measuring phase ambiguity based on two phase difference observation models. Section 4 presents a comparison among the existing phase-based methods and the proposed algorithms in terms of array aperture and computational complexity. In Section 5, simulations are performed to validate the performance of our methods. We conclude this paper in Section 6.

## 2. Signal Model

**Figure 1:**
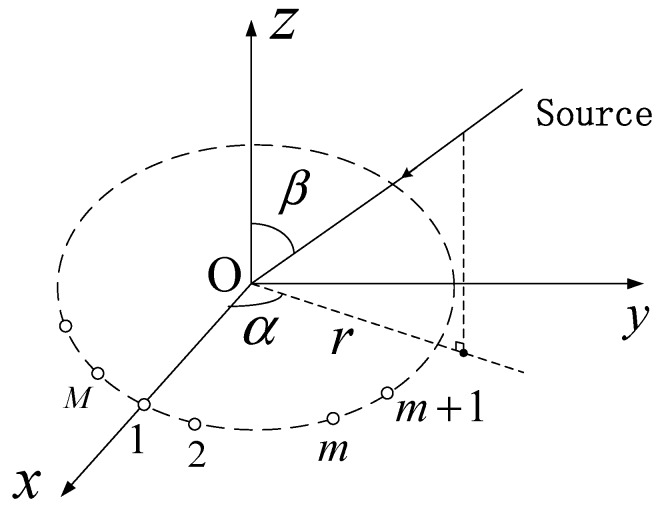
Signal model.

Consider a narrowband signal impinging on a UCA with radius r and M isotropic sensors uniformly distributed over the circumference in the xy-plane. Its geometry is depicted in Figure 1. The source is located at (α,β), where the azimuth angle α∈[−π,π) is measured counterclockwise from the x-axis, and the elevation angle β∈[0,π/2) is measured downward from the z-axis.

The output observation vector of the m th sensor at time index t has the following form

(1) $$x_{m}\left(t\right)=s\left(t\right)e^{j2\pi r/\lambda \cos\left(\alpha -\gamma_{m}\right)\sin\beta }+n_{m}\left(t\right)\;$$

where s(t) is assumed to be a zero-mean complex envelope with power $\sigma_{s}^{2}$, $n_{m}\left(t\right)$ represents the zero-mean white complex Gaussian noise with power $\sigma_{n}^{2}$, and $n_{m}\left(t\right)$ is spatially and temporally independent of s(t). λ is the source wavelength and $\gamma_{m}=2\pi \left(m-1\right)/M$ is the angle of the m th sensor measured counterclockwise from the x-axis.

In a matrix form, the array output vector x(t) can be modeled as

(2) $$\begin{array}{l} x\left(t\right)={\left[x_{1}\left(t\right),x_{2}\left(t\right),\cdots ,x_{M}\left(t\right)\right]}^{T}\\ \;=a\left(\alpha ,\beta \right)s\left(t\right)+n\left(t\right) \end{array}\;$$

where ${\left(\cdot \right)}^{T}$denotes the transpose operator, n(t) is the sensor noise vector, and a(α,β) is the M×1 steering vector corresponding to the DOA of the source, which can be written as

(3) $$a\left(\alpha ,\beta \right)=e^{j\left(2\pi r/\lambda \right)\cos\left(\alpha -\gamma_{m}\right)\sin\beta }.$$

The task of source localization is to estimate the azimuth and elevation angles of the source from a set of array observations $\left\{x_{k}\left(t\right)\right\}$. It can be seen that the exponent of (1) is represented by the 2-D angle parameters of the source. Therefore, one can estimate the source location from the phase differences of the received data.

## 3. Ambiguity Resolution of the Source’s Angles

To estimate the 2-D angles of the source, we first analyze the varying regularity of the phase differences between adjacent array elements of a UCA. In the absence of noise, by utilizing (1), the phase difference of the data received by the m th and m+1 th sensor can be derived as

(4) $$\phi_{m}=\frac{2\pi d}{\lambda }\sin\beta \sin\left(\left(2m-1\right)\frac{\pi }{M}-\alpha \right)\;$$

for m=1,2,⋯,M, and d=2rsinπ/M.

Equation (4) indicates that the unambiguous phase difference between adjacent array elements can be viewed as the samples of a standard one-period sinusoidal curve with zero baseline at time t=2m−1, whose amplitude and initial phase rely on the elevation and azimuth angle of the source, respectively. If we can obtain the unambiguous phase differences sequences $\left\{\phi_{m},m=1,2,\cdots ,M\right\}$, the source’s angle parameters can be estimated by using the LS algorithm [7,8]. Unfortunately, as previously mentioned, the measured phase differences will suffer from a serious phase ambiguity problem, which will lead to inaccuracies in the source’s parameter estimation when the array aperture is larger than the source’s half-wavelength. In this section, we first estimate the ambiguous phase differences between adjacent array elements of the UCA. Then, based on two phase difference observation models, two corresponding methods are proposed to resolve the problem of phase ambiguity, and the proof that the unambiguous DOA estimation can be achieved is given. Finally, the unambiguous closed-form angle estimates are determined via the LS algorithm by using the unambiguous phase differences.

### 3.1. Phase Difference Estimation

In [7,8], the phase differences are measured by utilizing the autocorrelation of $\left\{x_{m}\left(t\right)\right\}$

(5) $$u_{p,q}=\arg\left(R_{p,q}\right)\;$$

where $R_{p,q}=\frac{1}{K}\sum_{t=1}^{K}x_{p}\left(t\right)x_{q}^{*}\left(t\right)$, ${\left(\cdot \right)}^{*}$ denotes the complex conjugate, K is the number of snapshots, and $x_{p}\left(t\right)$ and $x_{q}\left(t\right)$ are the outputs of the p th and q th sensor, respectively.

It is worth noting that when the phase differences are measured in the time domain, the measured error of the phase differences will increase under the low signal-to-noise ratio (SNR) case. To improve the estimation accuracy, we measure the phase differences by using the signal subspace, which can lead to a higher SNR gain in the phase difference estimation. As we know, in the case of a single source, assuming that the vector of the signal subspace is μ, the following relationship holds

(6) μ=ca 

where c is a complex constant. This indicates that the characteristic of the phase difference can be represented by using the signal subspace μ. Then, the measured phase difference between the m th and m+1 th sensor of the UCA can be described as

(7) $${\tilde{\phi }}_{m}=\arg\left(\mu_{m}/\mu_{m+1}\right)\;$$

where $\mu_{m}$ and $\mu_{m+1}$ denote the m th and m+1 th entry of the signal subspace vector μ, respectively.

To avoid the high computation complexity caused by the eigenvalue decomposition, we apply the method of subspace tracking to obtain the signal subspace, which is not only free of eigenvalue decomposition but can also achieve a similar estimation accuracy to the method of taking the eigenvalue decomposition. The interested reader is referred to [19] for the solutions.

It should be noted that if the array radius r>λ/4sin(π/M), periodical ambiguities will be introduced into the measured phase differences, and the following relationship between the actual phase difference $\phi_{m}$ and the measured ${\tilde{\phi }}_{m}$ is established

(8) $$\phi_{m}={\tilde{\phi }}_{m}+2n\pi \;$$

where n is the number of 2π ambiguity periods.

In [7,8], the condition r≤λ/4 is commonly demanded to guarantee no phase ambiguity, which may be impractical in engineering applications. In the following subsections, we consider unambiguous DOA estimation under the case of the UCA with array radius r>λ/4, which is more practical in engineering applications.

### 3.2. Ambiguity Resolution Method under the Unambiguous-Relative Phase Observation Model

First, we define the phase difference observation model as the unambiguous-relative phase observation model, with the increment of the adjacent measured phase differences being less than π

(9) $$\left|{\tilde{\phi }}_{m+1}-{\tilde{\phi }}_{m}\right|\leq \pi .$$

Under the unambiguous-relative phase observation model, the condition that $r/\lambda \leq 1/8\sin^{2}\left(\pi /M\right)$ is needed to recover the unambiguous-relative phase differences by means of phase integral accumulation (refer to Appendix A).

Under this phase observation model, the following equation describes the process of recovering the unambiguous-relative phase differences

(10) $${\hat{\phi }}_{m}={\tilde{\phi }}_{1}+\sum_{i=2}^{m}\Delta {\tilde{\phi }}_{i}\;$$

where ${\hat{\phi }}_{m}$ denotes the recovered phase difference, and $\Delta {\tilde{\phi }}_{i}$ is the increment of the measured phase difference, which can be expressed as

(11) $$\Delta {\tilde{\phi }}_{i}=\left\{ \begin{array}{ll} {\tilde{\phi }}_{i}-{\tilde{\phi }}_{i-1}, & \left|{\tilde{\phi }}_{i}-{\tilde{\phi }}_{i-1}\right|<\pi \\ -2\pi +\left({\tilde{\phi }}_{i}-{\tilde{\phi }}_{i-1}\right), & {\tilde{\phi }}_{i}-{\tilde{\phi }}_{i-1}>\pi \;\\ 2\pi +\left({\tilde{\phi }}_{i}-{\tilde{\phi }}_{i-1}\right), & {\tilde{\phi }}_{i}-{\tilde{\phi }}_{i-1}<-\pi \; \end{array}\right..$$

By utilizing (10), the recovered unambiguous-relative phase differences can be obtained, which can be written as

(12) $${\hat{\phi }}_{m}=\frac{2\pi d}{\lambda }\sin\beta \sin\left(\left(2m-1\right)\frac{\pi }{M}-\alpha \right)+2n\pi \;$$

where 2nπ denotes the ambiguous phase introduced by the measured phase ${\tilde{\phi }}_{1}$.

The expression of ${\hat{\phi }}_{m}$ can be rewritten in the following form

(13) $${\hat{\phi }}_{m}=\frac{2\pi d}{\lambda }\left[\begin{array}{ccc} \sin\left(2m-1\right)\frac{\pi }{M} & \cos\left(2m-1\right)\frac{\pi }{M} & 1 \end{array}\right]\left[\begin{array}{c} \sin\beta \cos\alpha \\ -\sin\beta \sin\alpha \\ n\lambda /d \end{array}\right].$$

Expressing (13) in matrix form yields

(14) $$\hat{\Phi }=\mathrm{Ab}\;$$

where

(15) $$A=\left[\begin{array}{ccc} \sin\pi /M & \cos\pi /M & 1\\ \sin3\pi /M & \cos3\pi /M & 1\\ \vdots & \vdots & \vdots \\ \sin\left(2M-1\right)\pi /M & \cos\left(2M-1\right)\pi /M & 1 \end{array}\right]\;$$

(16) $$b=\frac{2\pi d}{\lambda }{\left[\begin{array}{ccc} \sin\beta \cos\alpha & -\sin\beta \sin\alpha & n\lambda /d \end{array}\right]}^{T}\;$$

and

(17) $$\hat{\Phi }={\left[\begin{array}{cccc} {\hat{\phi }}_{1} & {\hat{\phi }}_{2} & \cdots & {\hat{\phi }}_{M} \end{array}\right]}^{T}.$$

Then, the least squares estimation of b is given by

(18) $$b={\left(A^{T}A\right)}^{-1}a^{T}\hat{\Phi }.$$

The estimates of the azimuth and elevation angle can be determined as

(19) $$\alpha =angle\left(b_{1}-jb_{2}\right)\;$$

(20) $$\beta =\sin^{-1}\left(\frac{\lambda }{2\pi d}\sqrt{b_{1}^{2}+b_{2}^{2}}\right).$$

It can be seen that under the unambiguous-relative phase observation model, the unambiguous angle estimates can be obtained via the above procedure. However, if the array radius $r>\lambda /8\sin^{2}\left(\pi /M\right)$, the condition in (9) is not satisfied, and the method mentioned above cannot obtain the unambiguous DOA estimates. Then, one needs another way to solve the problem of phase ambiguity. In the next subsection, we present a novel algorithm for unambiguous angle estimation based on the parameter detection of a curve via the RHT algorithm.

### 3.3. Ambiguity Resolution Method under the Ambiguous-Relative Phase Observation Model

We define the phase difference observation model as the ambiguous-relative phase observation model with the increment of the adjacent measured phase differences satisfying the following relationship

(21) $$\left|{\tilde{\phi }}_{m+1}-{\tilde{\phi }}_{m}\right|>\pi .$$

As stated above, we cannot obtain the unambiguous-relative phase differences by the method of phase integral accumulation under this phase observation model. However, according to [20], we have known that a UCA with no less than six elements has no array ambiguity, which can be used to settle the problem of unambiguous DOA estimation under the spatial sub-Nyquist sampling.

The maximum ambiguity of the phase difference can be computed by

(22) D=⌈d/λ⌉ 

where ⌈⋅⌉ denotes the upper nearest integer. Then, based on the traverse of ambiguity, the phase difference matrix Φ is expressed as

(23) $$\Phi =\left[\begin{array}{cccc} {\tilde{\phi }}_{1}-2\pi D & {\tilde{\phi }}_{2}-2\pi D & \cdots & {\tilde{\phi }}_{M}-2\pi D\\ {\tilde{\phi }}_{1}-2\pi \left(D-1\right) & {\tilde{\phi }}_{2}-2\pi \left(D-1\right) & \cdots & {\tilde{\phi }}_{M}-2\pi \left(D-1\right)\\ \vdots & \vdots & \cdots & \vdots \\ {\tilde{\phi }}_{1}+2\pi D & {\tilde{\phi }}_{2}+2\pi D & \cdots & {\tilde{\phi }}_{M}+2\pi D \end{array}\right].$$

**Figure 2:**
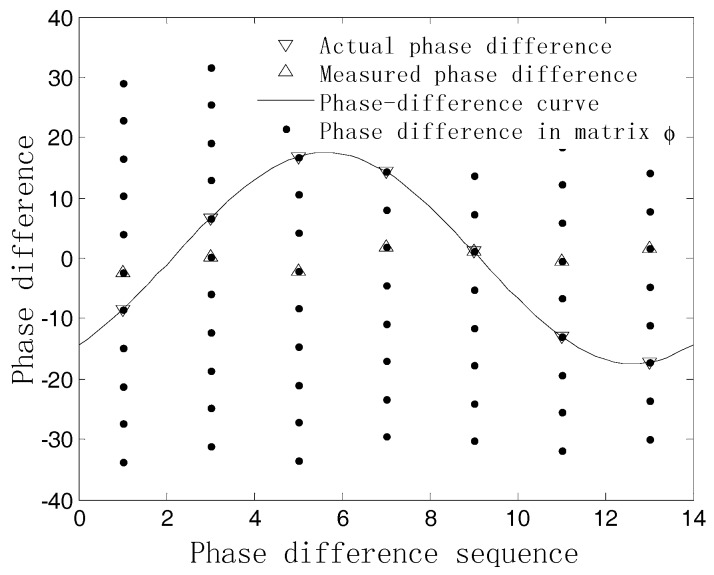
Phase difference relationship with M=7, array radius r=5λ.

We can see that each column in Φ includes an unambiguous phase difference, which can be viewed as the samples of the zero-baseline phase-difference curve. When mapping these phase differences into a phase-difference figure, the relationships between different kinds of phase differences can be depicted as in Figure 2. Thus, the 2-D angle information of the signal can be determined by detecting the zero-baseline sinusoidal curve via the randomized Hough transform.

Expressing (4) in the following form

(24) u=Asin(2πft+θ) 

where A=2πdsinβ/λ, f=1/2M, θ=−α, and t=2m−1, for m=1,2,⋯,M.

According to [21,22], the randomized Hough transform is usually used for curve detection in image processing. Its basic idea is to randomly pick n pixels from the image space and map them into one point in the parameter space. By extracting the cell of an accumulator with a local maximum of scores, the parameters of the detected curve can be estimated.

**Figure 3:**
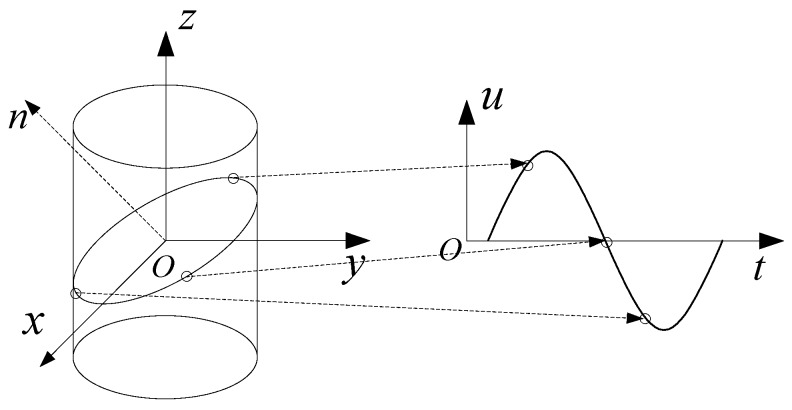
Mapping geometric relationship.

The mapping relations of the sinusoidal curve can be described by Figure 3. It can be seen that the intersection of a cylindrical surface and an inclined plane is an elliptic curve. When the cylindrical surface is extended along a generatrix of the cylinder, the elliptic curve becomes a sinusoidal curve. In other words, when the points on the sinusoidal curve are converted to the cylindrical surface, these points are all in the same plane.

According to [22], the mapping relationship between any point in the otu plane and the corresponding point on the cylinder surface can be written as

(25) $$\frac{t}{T}=\frac{\varphi -\pi }{-2\pi }\;$$

(26) $$x=R\cos\varphi =\frac{T}{2\pi }\cos\left[\pi \left(1-\frac{2t}{T}\right)\right]\;$$

(27) $$y=R\sin\varphi =\frac{T}{2\pi }\sin\left[\pi \left(1-\frac{2t}{T}\right)\right]\;$$

(28) z=u 

where T=2M is the period of the sinusoid, R is the radius of the cylindrical surface, and φ denotes the angle between the projection vector of a point on the oxy plane and the positive x axis.

Given any three points $\left(t_{i},u_{i}\right),\;i=1,2,3$ on the sinusoidal curve, their corresponding points on the cylindrical surface can be determined by Equations (25) to (28), which are represented as $\left(x_{i},y_{i},z_{i}\right),\;i=1,2,3$. These three points determine a plane whose normal vector is $n={\left(n_{x},n_{y},n_{z}\right)}^{T}$. Then, the following relationship holds

(29) A/R=tanγ 

where γ denotes the angle between the vector n and the positive z axis. Owing to the relationship $\cos\gamma =n_{z}$, the formula $\gamma =\arctan\sqrt{1-n_{z}^{2}}/n_{z}$ holds.

By utilizing Equations (24) to (29), the amplitude A and the initial phase θ can be determined

(30) $$A=\frac{T}{2\pi }\frac{\sqrt{1-n_{z}^{2}}}{n_{z}}\;$$

(31) $$\theta =\arcsin\left(\frac{u_{i}}{A}\right)-\frac{2\pi }{T}t_{i}.$$

Thus, the estimates of azimuth angle $\hat{\alpha }$ and elevation angle $\hat{\beta }$ can be written as

(32) $$\hat{\alpha }=\frac{2\pi }{T}t_{i}-\arcsin\left(\frac{u_{i}}{A}\right)\;$$

(33) $$\hat{\beta }=\arcsin\left(\frac{A\lambda }{2\pi d}\right).$$

Actually, the phase difference matrix Φ contains both the unambiguous phase differences and the ambiguous ones. If the selected points $\left(t_{i},u_{i}\right),\;i=1,2,3$ are samples of the actual phase-difference curve, the volume of the tetrahedron composed by the points $\left(x_{i},y_{i},z_{i}\right),\;i=1,2,3$ and the coordinate origin O is zero, since they are in the same plane. Thus, the following constraints are given to guarantee that the three points are selected from the zero-baseline sinusoidal curve, i.e., the unambiguous phase differences

(34) $$V=\left|\frac{T^{2}\left[u_{1}\sin\left(2\pi \left(t_{2}-t_{3}\right)/T\right)+u_{2}\sin\left(2\pi \left(t_{3}-t_{1}\right)/T\right)+u_{3}\sin\left(2\pi \left(t_{1}-t_{2}\right)/T\right)\right]}{16\pi^{2}}\right|\;$$

(35) $$E=\sum_{i=1}^{3}{\left[u_{i}-A\sin\left(2\pi ft_{i}+\theta \right)\right]}^{2}.$$

By minimizing (34) and (35), the three points can be bound to the zero-baseline sinusoidal curve.

Based on the above analyses, the implementation of the 2-D angle estimation by using the RHT algorithm can be summarized as follows:

Step 1. Construct the phase difference matrix Φ. Then, initialize a parameter data set Q=null and p=0.

Step 2. Randomly pick three columns $t_{i},\;i=1,2,3$ out of Φ in such a way that all columns in Φ have an equal probability to be taken.

Step 3. Traverse all the combinations of the phase differences in the three selected columns. Pick three points $\left(t_{i},u_{i}\right),\;i=1,2,3$ each time and calculate their corresponding points $\left(x_{i},y_{i},z_{i}\right),\;i=1,2,3$ according to Equations (25) to (28).

Step 4. Calculate the volume of the tetrahedron V and error function E according to Equations (34) and (35), respectively.

Step 5. If the volume $V<\delta_{v}$ and error function $E<\delta_{e}$, with $\delta_{v}$ and $\delta_{e}$ being two given tolerances, go to Step 6; otherwise, go to Step 3.

Step 6. Calculate the estimates of $\hat{\alpha }$ and $\hat{\beta }$ according to Equations (29) to (33), and determine one parameter point $q=\left(\hat{\alpha },\hat{\beta }\right)$.

Step 7. Search among set Q for an element $q_{c}$ such that $\left\|q_{c}-q\right\|<\delta$, with δ being a given tolerance. If found, go to Step 9; otherwise, go to Step 8.

Step 8. Attach to q an accumulating cell with score one, and insert it into set Q as a new element. Go to Step 10.

Step 9. Increase the score of the accumulating cell of $q_{c}$ by one. Go to Step 10.

Step 10. Check whether all the combinations of the phase differences in the three columns have been traversed; if yes, go to Step 11; otherwise, go to Step 3.

Step 11. p=p+1; if p>P then stop and go to Step 12; otherwise, go to Step 2.

Step 12. Search for the cell with the maximum scores. Its parameter coordinates are used to represent the 2-D angle estimates of the signal.

**Remark:** (1) The tolerance δ is introduced to further reduce the storage and adjust the resolution of the parameter space. When δ=0, the RHT has the highest resolution. The larger δ is, the lower the resolution is, but the less storage is used.

(2) P is a given number that denotes the number of times that we randomly pick three columns from the phase difference matrix Φ. In performing the RHT algorithm, the procedure of traversing all the combinations of the phase differences in the selected three columns is required to guarantee that the unambiguous phase differences can be picked every time.

It is worth noting that only three phase differences are used to estimate $\hat{\alpha }$ and $\hat{\beta }$ each time, and we call them the coarse DOA estimation. To improve the estimation accuracy, the coarse DOA estimates are used to resolve the problem of phase ambiguity. Then, the fine estimates of the 2-D angles can be determined by using the unambiguous phase differences.

Substitute the coarse DOA estimates $\hat{\alpha }$ and $\hat{\beta }$ into (4), and the phase-differences sequences $\left\{{\phi^{\prime }}_{m},m=1,2,\cdots ,M\right\}$ can be obtained. Then, the unambiguity phase differences in Φ can be picked as follows

(36) $$\begin{array}{l} k=\arg\;\underset{}{\min}\left|{\phi^{\prime }}_{m}-\Phi_{k,m}\right|\\ {\hat{\phi }}_{m}=\Phi_{k,m} \end{array}\}\;$$

for k=1,2,⋯,2D+1 denotes the different rows of the matrix Φ, and m=1,2,⋯,M represents different columns of the matrix Φ.

Suppose that the unambiguous phase differences in Φ are picked correctly and expressed in matrix form

(37) $$\hat{\Phi }={\left[\begin{array}{cccc} {\hat{\phi }}_{1}, & {\hat{\phi }}_{2}, & \cdots , & {\hat{\phi }}_{M} \end{array}\right]}^{T}.$$

Expressing (4) in matrix form yields

(38) $$\hat{\Phi }=A^{\prime }b^{\prime }\;$$

where

(39) $$A^{\prime }=\left[\begin{array}{cc} \sin\pi /M & \cos\pi /M\\ \sin3\pi /M & \cos3\pi /M\\ \vdots & \vdots \\ \sin\left(2M-1\right)\pi /M & \cos\left(2M-1\right)\pi /M \end{array}\right]\;$$

(40) $$b^{\prime }=\frac{2\pi d}{\lambda }{\left[\begin{array}{cc} \sin\beta \cos\alpha & -\sin\beta \sin\alpha \end{array}\right]}^{T}.$$

Then, the least squares estimate of $b^{\prime }$ is given by

(41) $$b^{\prime }={\left({A^{\prime }}^{T}A^{\prime }\right)}^{-1}{A^{\prime }}^{T}\hat{\Phi }.$$

The fine estimates of the azimuth and elevation angles can be determined as

(42) $$\alpha =angle\left(b_{1}-jb_{2}\right)\;$$

(43) $$\beta =\sin^{-1}\left(\frac{\lambda }{2\pi d}\sqrt{b_{1}^{2}+b_{2}^{2}}\right).$$

## 4. Discussion

In this section, we compare the proposed algorithm with two existing phase-based DOA estimation methods in [7,8]. We discuss these methods from the following aspects:

(1) Array aperture: With regards to the unambiguous DOA estimation, r≤λ/4 is strictly required in the previous method [7,8]. However, the proposed algorithm requires $r\leq \lambda /8\sin^{2}\left(\pi /M\right)$ under the unambiguous-relative phase observation model. With the condition sensor number M>6, there is no array ambiguity under the ambiguous-relative phase observation model. For example, the array radius r of a UCA with M=15 elements can be extended to 2.89λ under the UARPOM. Furthermore, under the ARPOM, the array radius can be determined based on the practical engineering application. Such an advantage can greatly improve the DOA estimation performance.

(2) Computational complexity: Let M and K symbolize the element number and snapshot number, respectively. Regarding the major computational complexity, MK/2+Ο(M) and (M−n)K+Ο(M) multiplications are required in [7,8], respectively, to calculate the autocorrelation function and perform the LS algorithm with a constant 1≤n≤M−2. However, the proposed algorithm under the UARPOM requires Ο(MK) multiplications to find the signal subspace by using the method of subspace tracking, Ο(M) additions in recovering the unambiguous-relative phase differences, and Ο(M) multiplications in the LS procedure. Likewise, the proposed algorithm under the ARPOM requires Ο(MK)+Ο(M) multiplications to find the signal subspace and perform the LS algorithm. In the coarse DOA estimation, suppose the number of randomly picked three column sets in Φ is P, then $Ο\left(P{\left(2D+1\right)}^{3}\right)$ multiplications are required to perform the RHT algorithm. The comparison results are listed in Table 1. Therefore, the computational complexity of the proposed algorithms is higher than that of methods in [7,8]. However, it is important to note that the proposed algorithms are more flexible for the selection of an array radius, which is more practical in the application of ambiguity resolution.

## 5. Simulation Results

In this section, several numerical simulations are conducted to validate the performance of the proposed algorithm and support the theoretical results relative to the previous method in [8], which is a generalized phase-based algorithm for the 2-D angle estimation of a single source with UCAs. Moreover, the Cramer–Rao lower Bounds (CRLBs) are also provided for comparison. In the following simulations, a single source located at (α,β)=(55.5°,40.3°) is considered. The performance is measured mainly in terms of spatial spectrum and root mean-square error (RMSE). The RMSE is defined as

(44) $$\mathrm{RMSE}=\sqrt{\frac{1}{M_{C}}\sum_{l=1}^{M_{C}}{\left({\hat{y}}_{l}-y\right)}^{2}}\;$$

where y stands for the parameters, such as α and β, ${\hat{y}}_{l}$ denotes the estimation of y in the l th trial, and $M_{C}$ is the number of Monte Carlo runs.

**Figure 4:**
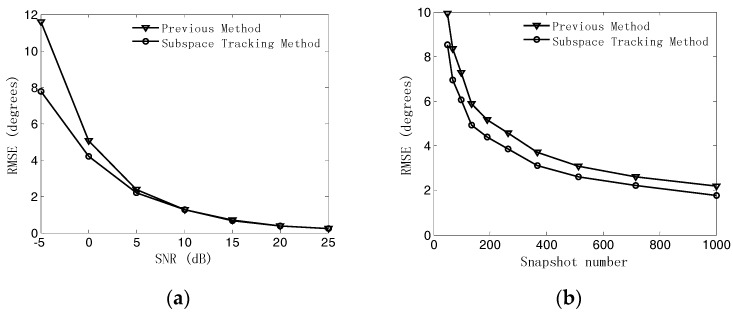
Root mean-square errors (RMSEs) of phase difference estimates. (**a**) RMSEs versus signal-to-noise ratio (SNR); (**b**) RMSEs versus snapshot number.

In the first experiment, we study the estimation accuracy of the measured phase difference by using the method of subspace tracking compared with the method in [8], which measures the phase difference in the time domain. The UCA with M=15 sensors and array radius r=λ/4 is considered. Figure 4a shows the RMSEs of the measured phase difference as a function of the SNR when the snapshot number is fixed at 200. Meanwhile, Figure 4b illustrates the RMSEs of the measured phase difference as a function of the snapshot number when the SNR is set equal to 0 dB. It can be seen that the estimation accuracy of the subspace tracking algorithm outperforms that of the previous method measured in the time domain, especially in the low SNR region and the small snapshot number case. Thus, our proposed algorithm will achieve a better performance in DOA estimation accuracy than the previous method.

**Figure 5:**
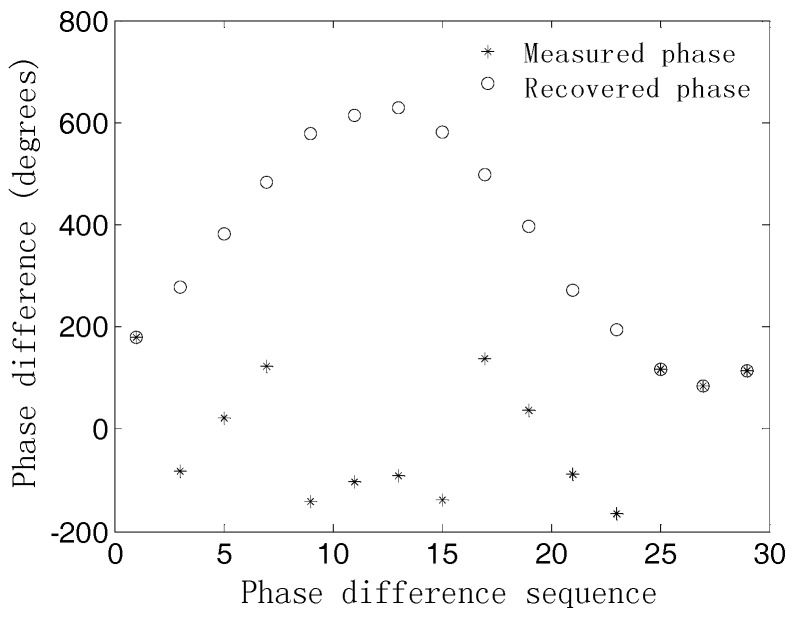
Measured phase differences and the recovered ones.

**Figure 6:**
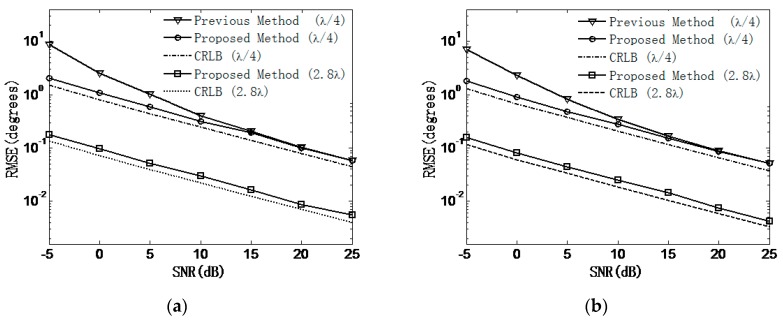
RMSEs of direction-of-arrival (DOA) estimates versus SNR. (**a**) Azimuth angle; (**b**) Elevation angle. CRLB, Cramer–Rao lower Bound.

In the second experiment, we study the estimation accuracy of our proposed algorithm under the unambiguous-relative phase observation model. First, the measured phase differences and the recovered ones are given to show that validity of our proposed algorithm. Then, the RMSEs of DOA estimates versus SNR are explored and compared to the results when using [8] and CRBLs [23]. We consider a UCA composed of M=15 elements with array radii r=λ/4 and r=2.8λ. However, for the method in [8], only r=λ/4 is simulated on account of the phase ambiguity problem. The other simulation parameters are the same as in the first experiment. Figure 5 and Figure 6 illustrate the recovered phase differences and the RMSEs of the 2-D DOA estimates of our proposed algorithm, respectively. It can be seen from Figure 5 that by using the method of phase integral accumulation, the recovered unambiguous-relative phase differences can be obtained. As shown in Figure 6a,b, it is clear that the estimation accuracy of the proposed algorithm outperforms that of the method in [8] for the same array radius, especially in the low SNR region. This can also be explained from the fact that the estimation accuracy of the phase differences measured in the time domain will deteriorate in the low SNR case, while the method of subspace tracking can obtain a higher SNR gain in the estimates of the phase differences. At the high SNR region, the DOA estimation accuracy of the previous method [8] approaches that of our method. The performance of the proposed algorithm is obviously enhanced when extending the array radius to r=2.8λ. As a whole, the simulation results indicate that the estimation performance of our proposed method outperforms the method in [8] and is also sufficiently close to that of the CRLB for both array radiuses r over a wide range of SNRs.

**Figure 7:**
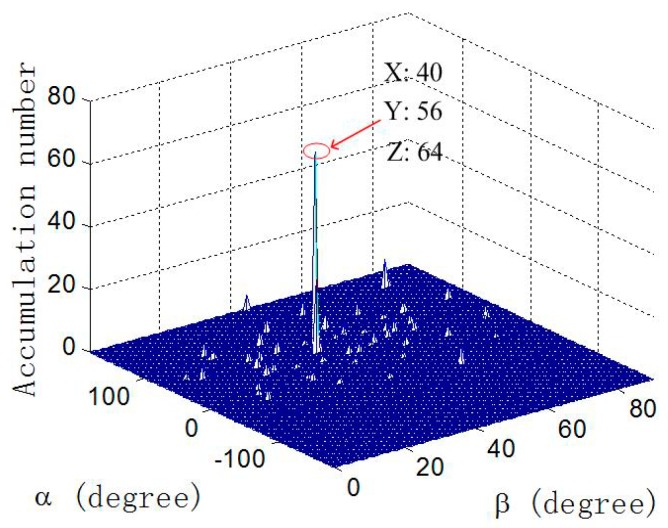
Coarse DOA estimation by the RHT algorithm with SNR = 10, array radius r=5λ.

**Figure 8:**
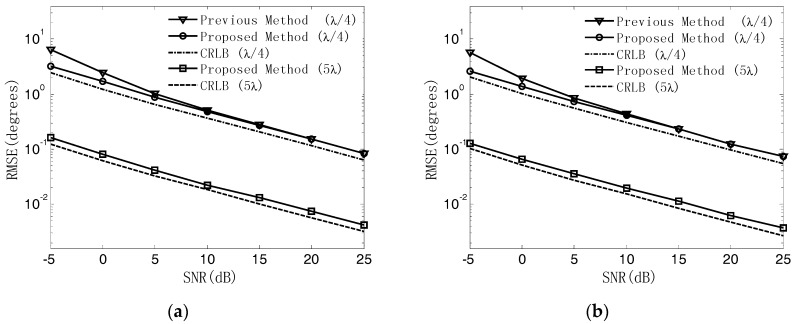
RMSEs of DOA estimates versus SNR. (**a**) Azimuth angle; (**b**) Elevation angle.

In the third experiment, the validity of our proposed algorithm under the ambiguous-relative phase observation model is verified, and the estimation accuracy is explored. First, the coarse estimates of the 2-D angles of the source are obtained by using the RHT algorithm. Then, by utilizing the coarse estimates of the DOA, the unambiguous phase differences can be obtained, with which the fine estimates of the 2-D angles of the source can be determined by the LS algorithm. Similar to the second experiment, the RMSEs of the DOA estimates versus SNR are explored and compared to the results when using [8] and CRBLs. The UCA composed of M=7 elements with array radii r=λ/4 and r=5λ is considered. The SNR varies from -5 dB to 25 dB in steps of 5 dB and the snapshot number is fixed at 200. At each SNR, 500 independent Monte Carlo trials are performed. The RHT algorithm is applied using the accumulating cell with a step size of (Δα,Δβ)=(1°,1°) and the given number P being 100. Figure 7 and Figure 8 illustrate the result of coarse DOA estimation by the RHT algorithm and the RMSEs of 2-D DOA estimates of our proposed method, respectively. It can be seen from Figure 7 that by setting an accumulating cell with a large step size, the coarse estimates of the 2-D angles can be obtained quickly and accurately, which is practical in engineering applications. Figure 8 indicates that the estimation performance of our proposed method outperforms the method in [8], and the accuracy of DOA estimation can be enhanced when extending the array radius to r=5λ.

**Figure 9:**
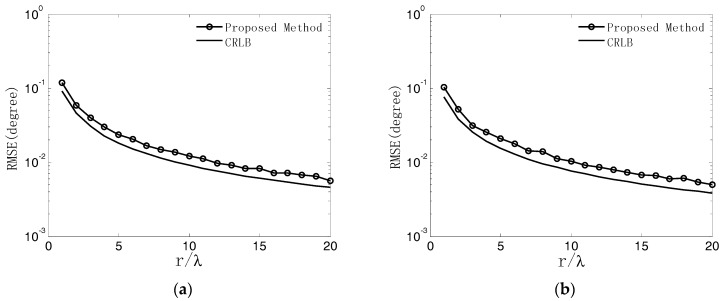
RMSEs of DOA estimates versus array radius. (**a**) Azimuth angle; (**b**) Elevation angle.

In the fourth experiment, the same parameters used in the third experiment are adopted except that the SNR is set equal to 10 dB, and the array radius varies from λ to 20λ. At each array radius, 500 independent Monte Carlo trials are performed. The RMSEs of DOA estimates are shown in Figure 9a,b. It is obvious that the RMSEs of the proposed algorithm decrease monotonically as the array radius increases. This is because a larger array radius will produce a better estimation of the phase differences.

**Figure 10:**
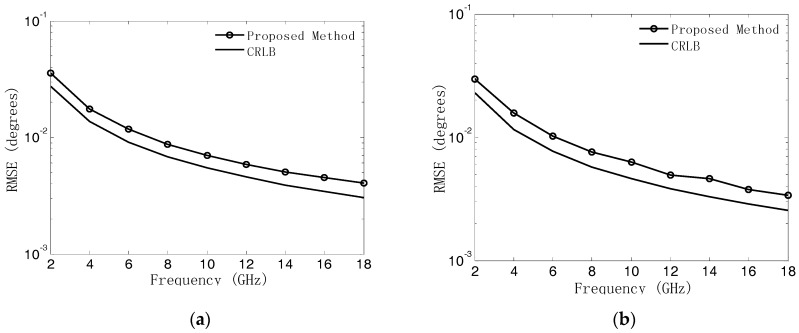
RMSEs of DOA estimates versus carrier frequency. (**a**) Azimuth angle; (**b**) Elevation angle.

In the last experiment, we verify the DOA estimation performance of our proposed algorithm under the ambiguous-relative phase observation model against the carrier frequency. The simulation conditions are the same as those of the third experiment except that SNR = 10 dB, the array radius r=0.5 m , and the carrier frequency varies from 2 GHz to 18 GHz in steps of 2 GHz. A total of 500 independent Monte Carlo trials have been conducted for every fixed carrier frequency. The RMSEs of DOA estimates are shown in Figure 10a,b. We can find that the proposed algorithm can obtain the unambiguous angle estimation in a wide frequency band, which is promising in practical applications, especially for an electronic warfare environment. Moreover, the RMSEs of the proposed algorithm decrease as the carrier frequency increases, which can be explained by the fact that the array aperture relatively becomes bigger as the carrier increases. Thus, the performance of the DOA estimation can be improved.

## 6. Conclusions

In this paper, two novel ambiguity resolution algorithms are proposed for a source’s 2-D parameter estimation using a UCA. Based on the two phase observation models, the DOA parameters have been determined without the operation of a multidimensional search. Compared with the existing phase-based methods, the proposed algorithms have the main advantage that they can greatly enhance the DOA estimation performance by extending the array radius. Such an advantage could be applied more broadly in practice than the previous methods of ambiguity resolution. Simulation results show the effectiveness in ambiguity resolution and the satisfactory estimation performance of our algorithms, especially when the SNR is low. However, some slight limitations also exist in our methods, including the high computational load in performing the RHT algorithm especially when the array radius is large.

## Figures and Tables

**Table 1:** Computational complexity comparison.

Algorithms	Statistical Matrices	Subspace Tracking	Recover Curve	RHT Algorithm	LS Algorithm
Method in [7]	MK/2	-	-	-	Ο(M)
Method in [8]	(M−n)K	-	-	-	Ο(M)
Method under UARPOM	-	Ο(MK)	Ο(M)	-	Ο(M)
Method under ARPOM	-	Ο(MK)	-	$Ο\left(P{\left(2D+1\right)}^{3}\right)$	Ο(M)

RHT, randomized Hough transform; LS, least squares.

## Appendix A

In this section, we will prove the condition that the unambiguous angle estimates of a source with any direction of arrival can be determined by using the measured phase differences under the unambiguous-relative phase observation model.

First, we define $\phi_{m}$ and $\phi_{n}$ as two disparate phase difference observations if the following equation holds

(A1) $$\left(\theta_{n}-\theta_{m}\right)\mathrm{mod}\pi \neq 0\;$$

where $\theta_{m}=\left(2m-1\right)\pi /M$ and $\theta_{n}=\left(2n-1\right)\pi /M$.

Under the unambiguous-relative phase observation model, the phase differences can be expressed as

(A2) $${\hat{\phi }}_{m}=\frac{2\pi d}{\lambda }\sin\beta \sin\left(\left(2m-1\right)\frac{\pi }{M}-\alpha \right)+2n\pi +\delta_{m}\;$$

where ${\hat{\phi }}_{m}$ denotes the unambiguous-relative phase difference to the first phase difference ${\hat{\phi }}_{1}$, and 2nπ is the ambiguous phase.

We can see from Equation (A2) that if the number of the disparate phase-difference observations is no less than three, the 2-D angles of the source and ambiguity number n can be determined. Thus, by recovering the unambiguous-relative phase differences, the 2-D angles of the source can be determined by using the LS algorithm.

In the absence of noise, by using (4), the increment of the adjacent measured phase differences of the m th and m+1 th element can be derived as

(A3) $$\begin{array}{ll} \phi_{m+1}-\phi_{m} & =\frac{2\pi d}{\lambda }\sin\beta \sin\left(\left(2m+1\right)\frac{\pi }{M}-\alpha \right)-\frac{2\pi d}{\lambda }\sin\beta \sin\left(\left(2m-1\right)\frac{\pi }{M}-\alpha \right)\\ & =\frac{4\pi d}{\lambda }\sin\frac{\pi }{M}\sin\beta \cos\left(2m\frac{\pi }{M}-\alpha \right) \end{array}\;$$

where d=2rsinπ/M.

Substituting (A3) into (9) yields

(A4) $$\left|\frac{4\pi d}{\lambda }\sin\frac{\pi }{M}\sin\beta \cos\left(2m\frac{\pi }{M}-\alpha \right)\right|\leq \pi \;$$

Because $-1\leq \sin\beta \cos\left(2m\frac{\pi }{M}-\alpha \right)\leq 1$, the following equation holds

(A5) $$\frac{4\pi d}{\lambda }\sin\frac{\pi }{M}\leq \pi .$$

Substituting d=2rsinπ/M into (A5), the relationship between the radius r and the number of elements M of the UCA can be obtained as

(A6) $$r\leq \frac{\lambda }{8\sin^{2}\left(\pi /M\right)}.$$
